# Neutrophil Dynamics Contribute to Disease Progression and Poor Survival in Pancreatic Cancer

**DOI:** 10.3390/cancers17213541

**Published:** 2025-11-01

**Authors:** Reegan Sturgeon, Paran Goel, Caitlin Molczyk, Ridhi Bhola, Paul M. Grandgenett, Michael A. Hollingsworth, Rakesh K. Singh

**Affiliations:** 1 Department of Pathology, Microbiology, and Immunology, The University of Nebraska Medical Center, Omaha, NE 68198, USA; rsturgeon@unmc.edu (R.S.); futuredoc.caitlin@gmail.com (C.M.); rbhola@unmc.edu (R.B.); 2 Division of Hematology and Oncology, The University of Alabama, Birmingham, AL 35294, USA; goelp@uab.edu; 3 Eppley Institute for Research in Cancer and Allied Diseases, Fred & Pamela Buffett Cancer Center, University of Nebraska Medical Center, Omaha, NE 68198, USAdhollingsworth@unmc.edu (M.A.H.)

**Keywords:** cancer immunology, tumor-associated neutrophils, pancreatic cancer, CXCR2, tumor microenvironment

## Abstract

**Background**: Tumor-promoting inflammation and immune evasion are hallmarks of cancer, contributing to the survival and proliferation of tumor cells. Infiltrating leukocytes and pro-inflammatory cytokines released into the tumor microenvironment (TME) often cause this inflammation and immune evasion. Neutrophils are leukocytes that contribute to inflammation and have immunomodulatory functions. They are shown to have pro- or anti-tumorigenic roles contingent on cancer type. **Methods**: In this study, we examined the role of neutrophil recruitment in pancreatic cancer (PC) progression using patient samples and murine models. **Results**: We observed enhanced neutrophil infiltration and neutrophil extracellular trap (NET) formation, which were dependent on disease stage and tumor site. Our murine model studies showed that the infiltration of neutrophils and NETs was dependent on disease progression. Moreover, chemokine receptor CXCR2 and its ligands played a crucial role in neutrophil recruitment. The expression of CXCR2 and its ligands was associated with a worse prognosis for patients. **Conclusions**: Our data demonstrates that gemcitabine therapy enhances neutrophil recruitment and NET formation in PC. In addition, we observed altered neutrophil phenotypes in PC dependent on disease progression, suggesting a context-dependent immunomodulatory role. Together, our data demonstrate that CXCR2-driven neutrophil recruitment increases with PC progression, is enhanced by gemcitabine chemotherapy, promotes an immunosuppressive microenvironment, and is associated with poor patient survival.

## Simple Summary

The present study investigates how neutrophil recruitment and neutrophil extracellular trap (NET) formation influence pancreatic cancer progression and patient prognosis. Neutrophil infiltration and NET formation increase with disease progression. CXCR2 and its ligands drive neutrophil recruitment and high CXCR2 expression correlates with poor prognosis. Gemcitabine therapy (a standard chemotherapy for PC) enhances neutrophil recruitment and NET formation. Neutrophil phenotypes shift with disease progression, suggesting context-dependent immunomodulatory roles. Overall, CXCR2-driven neutrophil activity promotes an immunosuppressive TME and contributes to pancreatic cancer progression.

## 1. Introduction

With a mere 13% 5-year survival rate, PC is quickly becoming one of the deadliest forms of cancer [1]. Treatment options are limited, and this can be attributed to many factors, such as late-stage diagnosis, metastasis, and/or an inflammatory and immunosuppressive tumor microenvironment (TME) [2,3,4,5,6,7,8,9,10,11]. Tumor-promoting inflammation is a hallmark of cancer, contributing to malignant cells’ survival and proliferation [12]. Avoiding immune destruction is another hallmark of cancer, and one of the ways tumors do this is by creating an immunosuppressive TME [5,12]. Infiltrating leukocytes and pro-inflammatory cytokines released into the TME are often the cause of this inflammation and immunosuppression [13,14,15,16]. One of the leukocytes shown to contribute to this environment is the neutrophil [14,17,18,19,20].

Neutrophils are the most abundant immune cells in the body, making up roughly 50–70% of white blood cells, with a short half-life of less than 24 hrs [21,22]. Their role in the normal body function is to control inflammation and defend against extracellular pathogens, whether that defense is through exterminating the pathogen itself or signaling for additional aid from other immune cells [23]. Neutrophil functions are often regulated by the manner in which they undergo cell death, which involves specific cell death pathways, including apoptosis, pyroptosis, ferroptosis, necroptosis, and necrosis [21,22]. Their death is so important that they have their own form of cell death, NETosis. When neutrophils are too small to phagocytose a pathogen, they may opt to undergo this special type of neutrophil cell death, which is unique from other cell deaths due to the formation of neutrophil extracellular traps (NETs). NETs are a web-like structure made from a mixture of DNA, histones, antimicrobial granules, and neutrophil elastase that damage and contain a pathogen until it can be killed by another immune cell [21,22,24]. Although short-lived, neutrophils play a crucial role in pathogen defense; however, under certain circumstances, they can be detrimental to patient health.

Initially thought to only bind to pathogens, impairing their movement and eventually degrading them, the past 15 years have shed some light on their involvement in various inflammatory diseases, such as cancer [25]. An abundance of various neutrophil activators in the TME, such as CXCL8 and CXCR1/CXCR2 ligands, often creates a hospitable environment for NET formation [26,27]. Like their precursor neutrophils, NETs have been implicated in both pro-tumor and anti-tumorigenic capabilities [25]. However, in the context of PDAC, NETs are considered pro-tumorigenic [28,29]. They were shown to orchestrate circulating tumor cell extravasation by binding to them and escorting them to the site of metastasis [24,28,30,31,32,33,34]. They have also been shown to aid in the setup of a pre-metastatic niche [25,31,33,34]. This partnership between PDAC and NETs also serves as a mechanical barrier between tumor cells and other cytotoxic immune cells like natural killer cells and cytotoxic T-cells, preventing physical contact between them and tumor cell degradation [27,28,29,32,34,35].

Neutrophils have been evaluated as a prognostic indicator of patient survival in PC, especially in the context of being compared to circulating lymphocytes, the neutrophil to lymphocyte ratio (NLR) [36]. A high NLR in PC patients is a prognostic indicator of worse survival [36]. They contribute to the very complex TME and desmoplasia surrounding the tumor of PC patients, which is both immunosuppressive and highly inflammatory [37,38,39,40]. Not only do neutrophils contribute to the primary tumor, but they are also implicated in metastasis, as NETs are essential to PC metastasis [25,31,33,34]. Even reactive oxygen species (ROS) have been implicated in PC development and metastasis, dependent on concentration [41,42]. In this study, we examined the role of neutrophil recruitment in PC progression using patient samples and murine models. This study reveals that CXCR2-mediated neutrophil infiltration and NET formation escalate with pancreatic cancer progression, are enhanced by gemcitabine chemotherapy, contribute to an immunosuppressive microenvironment, and are linked to reduced patient survival.

## 2. Materials and Methods

### 2.1. Human Tumor Specimens and Animal Models

KC, Pdx1-cre; LSL-KrasG12D, KCC, Pdx1-cre; LSL-KrasG12D; Cxcr2^−/−^, and KPC mice, LSL-KrasG12D/+, LSL-Trp53R172H/+, and Pdx-1-Cre, were bred in the University of Nebraska Medical Center (UNMC) animal facility, a gift from the Batra lab, who developed the mouse strains [43,44]. The mice were sacrificed at relevant time points, 10–50 weeks and 5–25 weeks, respectively, with a control group that did not form tumors sacrificed at the experiment endpoint. Animals used for these studies will be euthanized by CO_2_ inhalation using AVMA guidelines for euthanasia. This study contained no gender bias, so both genders of mice were used. Pancreases and tumors were removed from the mice and sent to the UNMC core facility for slicing and slide mounting.

Tissue microarray (TMA) slides were obtained from different sources. The TMA obtained from the University of Nebraska Medical Center (UNMC) Rapid Autopsy Program was constructed from paraffin blocks containing 21 PC tumor cases, three non-matched normal pancreases, and 32 tissue cores from different metastatic sites, such as liver, diaphragm, and others. The Institutional Review Board of the UNMC approved this study (UNMC IRB 091-01). Another TMA of PC (Accumax Array, Republic of Korea A207III), containing thirty-three cases and eight unmatched normal pancreatic tissues in duplicate, was a generous gift from Petagene (Seoul, Republic of Korea).

### 2.2. Immunostaining and Quantitation

For immunofluorescent images, fixed paraffin-embedded tissue samples on slides were deparaffinized in a series of xylene and alcohol treatments (two times xylenes, one time each in 100%, 95%, 80%, and 70% alcohol) for 5 min each, then washed with DI water for 5 min. Antigen retrieval was performed using 10 mM sodium citrate buffer (pH = 6.0), microwaved in a pressure cooker on medium-high for 1 min and medium for 10 min to improve antigen retrieval. Sections were allowed to cool for 30 min, washed with DI water for 5 min, and then marked using a Hydrophobic Barrier Pap Pen. The slides were washed 3 times with TBS and the last wash was left on for 5 min. Three times and quenched for endogenous peroxidase activity by incubating tissue in 3% H_2_O_2_ in MilliQ water for 5 min. Slides were again washed thoroughly with TBS and blocked with antibody diluent reagent (BD Biosciences, Franklin Lakes, NJ, USA) for 30 min. Slides were blocked and then stained with primary antibody overnight at 4 °C. Primary antibodies are listed in Table 1. Slides were washed thoroughly with TBS. The slides were treated with a second antibody (listed in Table 1), either green fluorescent or biotinylated antibody (then treated with an avidin-bound fluorescent molecule), for 1 h at room temperature. Nuclei were stained using a DAPI flouromount (Southern Biotech, Birmingham, AL, USA). The slides were observed under a Nikon Eclipse E800 microscope at 200× magnification (Nikon, Melville, NY, USA, RRID: SCR_020326) and NIS-Elements BR 5.11.00 software (Nikon v5.11.00, RRID: SCR_014329). The signals were calculated per high-power frame from 3 to 5 pictures per mouse and patient sample. Images were processed via ImageJ-win64 to overlap DAPI and fluorescent images, and overlapping signals were counted as a positive mark for antibody presence. 3–5 patient samples were used per group for patient data.

To explore the expression of myeloperoxidase in the KC mouse model, we performed immunohistochemical (IHC) staining. After blocking non-specific binding by incubating with serum, slides were probed with respective primary antibodies (Table 1) and incubated overnight at 4 °C. The next morning, slides were washed and incubated with appropriate secondary antibodies. The ABC Elite Kit and DAB substrate kit (3, 3-diaminobenzidine) (Vector Laboratories, Burlingame, CA, USA) were used to detect immunoreactivity per the manufacturer’s protocols. A positive IHC staining gave a brown color (cytoplasmic, nuclear, membrane, or all, depending on the proteins’ location), while the nuclei were counterstained blue with Meyer’s Hematoxylin (Thermo Scientific, Fremont, CA, USA). The slides were observed under a Nikon Eclipse E800 microscope at 200× magnification (Nikon, Melville, NY, USA, RRID: SCR_020326) and NIS-Elements BR v5.11.00 software (Nikon v5.11.00, RRID: SCR_014329). The signals were calculated per high-power frame from 3 to 5 pictures per mouse. Quantitation of the signals was performed using ImageJ software, with individual signals being analyzed, counted, and confirmed by two independent observers.

### 2.3. Gene Expression Analysis

Gene expression data for pancreatic cancer patients, as well as normal and metastatic tissue samples, were retrieved from the TNMplot database (www.tnmplot.com, RRID: SCR_026319) [45]. This online resource provides comprehensive gene expression profiles from cohorts of patients from various cancer types, including pancreatic cancer, across various tissue types (tumor, normal, and metastatic). The database contains curated datasets from publicly available sources, enabling comparative analysis of gene expression in distinct tissue types and across disease stages.

Gene expression and clinical survival data for patients with PC were obtained from the Kaplan–Meier plotter (RRID: SCR_018753), an online resource that integrates publicly available patient samples with detailed gene expression profiles and clinical outcomes [46]. This database provides comprehensive information on gene expression levels at different time points and allows for survival analysis based on gene expression and clinical variables. The cohort included in the study consisted of patients diagnosed with PC, with available gene expression data for CXCR1, CXCR2, CXCL5, and CXCL8, along with survival information.

### 2.4. Statistical Analysis

Statistical analysis was conducted using GraphPad Prism 10 software (GraphPad Software, Inc., La Jolla, CA, USA). The significance was determined by Student’s *t*-test or Nonparametric Mann–Whitney U-test when two groups were being compared. One-way ANOVA was used for samples containing more than one group/sample per column. For all statistical tests, a *p*-value of ≤0.05 was considered significant, with more asterisks (*) indicating more significance (*p*-value = * ≤ 0.05, ** ≤ 0.01, *** ≤ 0.001, and **** ≤ 0.0001).

## 3. Results

### 3.1. Increased Neutrophil Infiltration and NETs with Disease Progression

**Figure 1:**
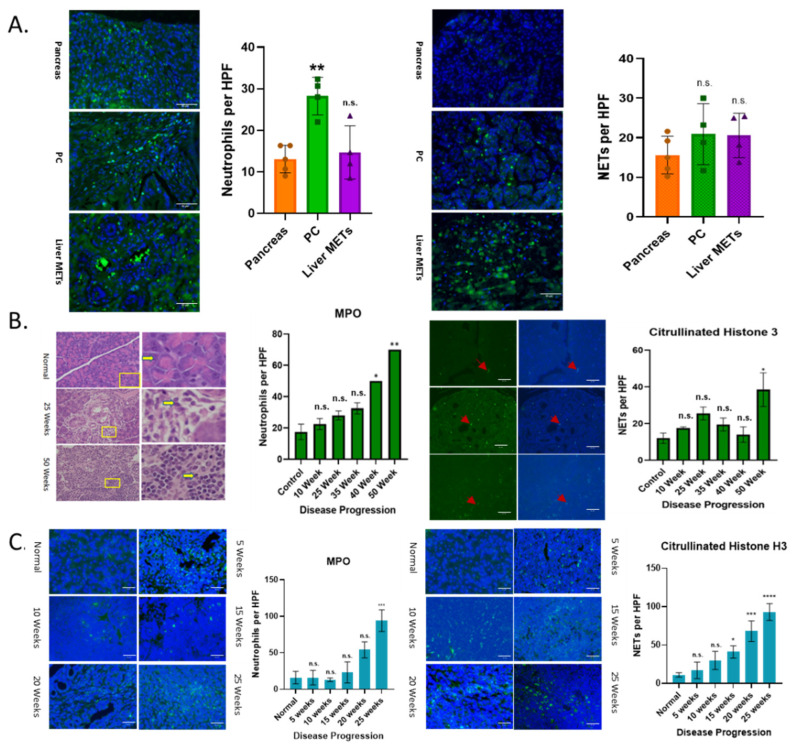
Increased neutrophil infiltration and activation with disease progression. Patient samples (n = 20) (**A**) and 2 mouse models KC (Pdx1-cre; LSL-KrasG12D) (n = 3–5) (**B**) and KPC (LSL-KrasG12D/+; LSL-Trp53R172H/+; Pdx-1-Cre) (n = 3) (**C**), were analyzed via IF to look for infiltrating neutrophils and NET formation using MPO and Citrullinated Histone H3, respectively. Patient samples show a statistically significant increase in the infiltration of neutrophils in primary PC tumors, with METs and normal pancreas having relatively similar amounts (**A**). NET formation was slightly higher in the primary tumor and METs in patient samples compared to the normal pancreas (**A**). Both KC and KPC mouse models demonstrated significantly higher infiltration of neutrophils and formation of NETs as the disease progressed (**B**,**C**). The arrows on **B** show examples of a positive signal. Statistical analysis was performed in GraphPad Pro (ver 10.4.2) via a One-way ANOVA. Number of asterisks associated with *p*-value (* ≤ 0.05, ** ≤ 0.01, *** ≤ 0.001, and **** ≤ 0.0001); n.s., non-significant.

Using animal models and human tumors, we have demonstrated a progressive increase in the expression of CXCR2 ligands (potent neutrophil chemoattractants) with the progression of PC disease [47]. We analyzed whether neutrophil recruitment was altered in human PC primary tumors and metastases (METs), and whether there is a dynamic increase in neutrophil recruitment through disease progression in animal models. Samples from patients’ primary PC tumors and liver MET tissues were analyzed for the presence of neutrophils. Primary tumors showed significantly elevated infiltration of neutrophils compared to normal patient samples (Figure 1A). Liver METs showed a slight increase in infiltration when compared to normal samples. Next, we analyzed for the presence of neutrophil extracellular traps (NETs) (Figure 1A). Both primary tumors and liver METs showed a trend toward a higher frequency of NETs compared with normal samples; however, these differences were not statistically significant. This is likely due to limited sample sizes and the fact that, at the time of diagnosis, patients often have metastasis and may not require as many NETs.

We wanted to investigate how the recruitment and activation of neutrophils change with disease progression using relevant mouse models of PC. We first looked at a Kras^G12D^ mutation, one of the most common PC mutations, through the KC mouse model (Pdx1-cre; LSL-KrasG12D) (Figure 1B). We observed increased neutrophil recruitment with disease progression, which was statistically significant at 40 and 50 weeks. Overall, there was an increase in the formation of NETs with disease progression (Figure 1B).

Next, we confirmed these findings using the KPC mouse model (LSL-KrasG12D/+; LSL-Trp53R172H/+; Pdx-1-Cre), which has a P53 mutation in addition to the Kras^G12D^ mutation. This is a more aggressive PC mouse model, with mice sacrificed at 25 weeks due to quick disease progression. Both neutrophil recruitment and NET formation showed statistically significant increases with disease progression (Figure 1C).

### 3.2. CXCR2 and Its Ligands Play an Important Role in Neutrophil Recruitment

**Figure 2:**
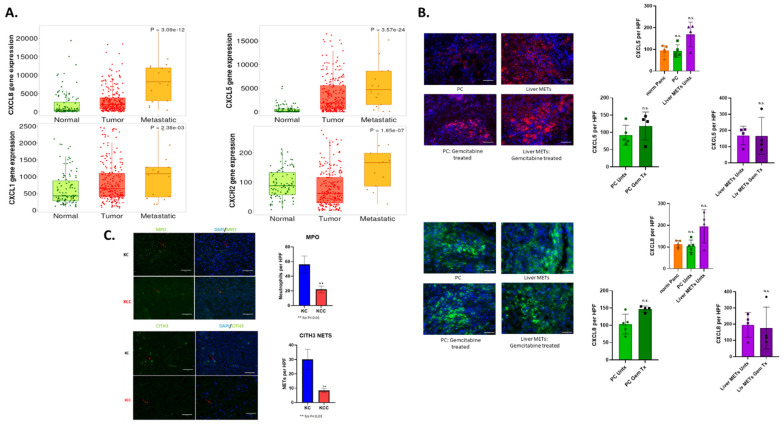
CXCR2 and its ligands are essential for neutrophil recruitment. TMNplot database was used to analyze the expression of genes from patient samples (normal, tumor, and METs) (n = 20) who were diagnosed with PC (**A**). Primary tumors and METs from patient samples have increased expression of CXCR2 and its ligands compared to normal pancreatic tissue, with METs showing the highest expression of these genes. We tested our patient samples for CXCL5 and 8 using immunofluorescence (**B**). Comparable to the TMNplots, there were increases in the expression of CXCL5 and CXCL8, primarily in the Liver METs and gemcitabine treatment, showing a trend towards increasing expression in primary tumors. KC mice (K-rasG12D; Pdx-1cre) had CXCR2 genetically KO to form KCC mice (K-rasG12D; Pdx-1cre; CXCR2−/+), and immunofluorescence was performed on the primary tumor to look at neutrophil recruitment and NET formation (**C**). CXCR2 KO moderately reduced neutrophil infiltration and NET formation in KCC mouse model when compared to KC mice. Statistical analysis was done in GraphPad Pro via a non-parametric Mann–Whitney *t*-test or One-Way ANOVA. Scale Bars: 50 µm. Number of asterisks associated with *p*-value, ** ≤ 0.01); n.s., non-significant.

CXCR2 and its associated ligands are one of the major recruiters of neutrophils to sites of infection and injury [48,49,50]. We have previously researched the role of the CXCR1/2 axis and its role in PC progression, patient prognosis, and development of chemotherapy resistance [51,52]. Therefore, we analyzed the expression of CXCR2 and its ligands in patient samples. We examined CXCR2, CXCL1, CXCL5, and CXCL8 in PC tumors and METs compared to normal pancreatic tissue (Figure 2A). We observed differential expressions of these genes when we looked at primary tumor data compared to normal tissue (Figure 2A). Expression of these genes is highly variable, dependent on individuals in both normal and primary tumors. However, when we examine metastatic samples, although there is high individual patient variability, a statistically significant increase is observed in all genes examined. We wanted to evaluate if CXCR2 ligands were expressed differently between primary and metastatic patient tumors and if this was affected by gemcitabine chemotherapy, the PC standard of care [53]. We used antibodies for CXCL5 and CXCL8, the two most highly expressed in PC MET TNMplot samples, via immunofluorescence to compare expression in untreated and gemcitabine treated samples from primary tumor and liver METs to normal pancreatic tissue samples (Figure 2B). Similarly to the PC patient expression data, there is not much difference in the expression of either ligand when comparing primary patient tumor and normal samples. However, while not seen to be statistically significant, both ligands showed enhanced expression in liver MET samples, again highlighting their role in metastasis. When we compare MET samples before and after gemcitabine treatment, we do not see any difference in CXCL5 or CXCL8 expression. There was, however, increased expression of these ligands in primary tumors post-Gemcitabine treatment compared to untreated tumors. This also indicates that, in addition to playing a role in metastasis, we hypothesize that neutrophils may also play a role in gemcitabine chemotherapy resistance in PC tumors.

To understand the relationship between the CXCR2 axis and neutrophils in primary PC tumors, we selectively knocked out (KO) Cxcr2 in KC mouse models, generating the KCC mouse model (Pdx1-cre; LSL-KrasG12D; Cxcr2^−/−^). Tumors from these mice were analyzed for the presence of neutrophils and NETs using immunofluorescence as previously described (Figure 2C). There are statistically significant decreases in the recruitment of neutrophils to the TME of KCC mice, as well as decreases in NET formation, demonstrating an important role of CXCR2 and its ligands in neutrophil recruitment in PC.

### 3.3. Chemotherapy Enhances Neutrophil Recruitment and NET Formation in Patient Samples

**Figure 3:**
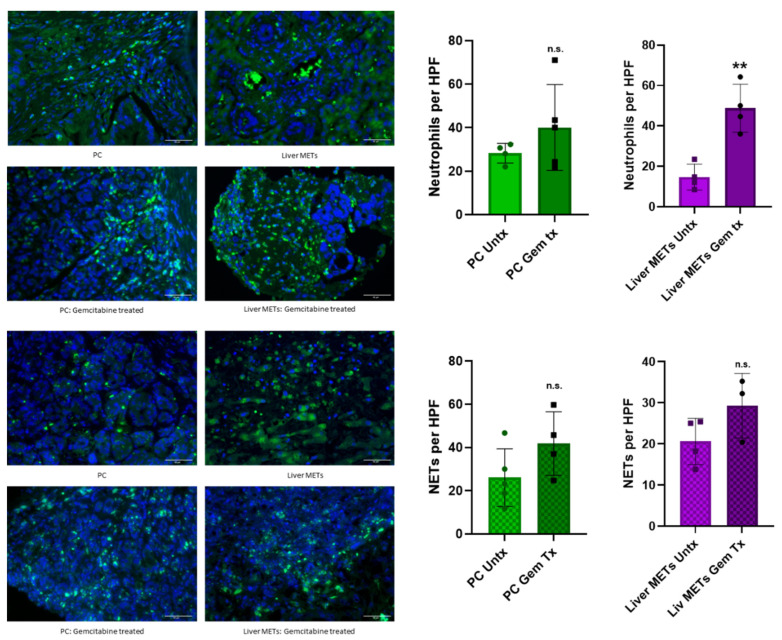
Gemcitabine treatment enhances neutrophil recruitment and activation in patient samples. Patient samples (n = 20) were analyzed via immunofluorescence for the presence of neutrophils and NETs. Primary tumors and METs were compared to their gemcitabine-treated counterpart. Both neutrophils and NETS were increased in gemcitabine-treated patient samples when compared to untreated samples, regardless of site. Statistical analysis was done in GraphPad Pro via a *t*-test. Scale Bars: 50 µm. *p*-value (** ≤ 0.01); n.s., non-significant.

Next, we examined whether gemcitabine chemotherapy affects neutrophil recruitment and activation. We analyzed patient samples from primary tumors and liver METs pre- and post-gemcitabine treatment for the infiltration of neutrophils and NETs (Figure 3). We observed an increase in the infiltration of neutrophils as well as the formation of NETs (Figure 3). There was also an increase in both neutrophils and NETs in the primary tumor in gemcitabine-treated patients compared to the untreated samples. These data indicate that gemcitabine chemotherapy enhances neutrophil recruitment and NET formation in PC. This data adds further weight to our previous hypothesis that neutrophils may contribute to the development of gemcitabine chemotherapy resistance.

### 3.4. Altered Neutrophil Phenotype in PC with Disease Progression

**Figure 4:**
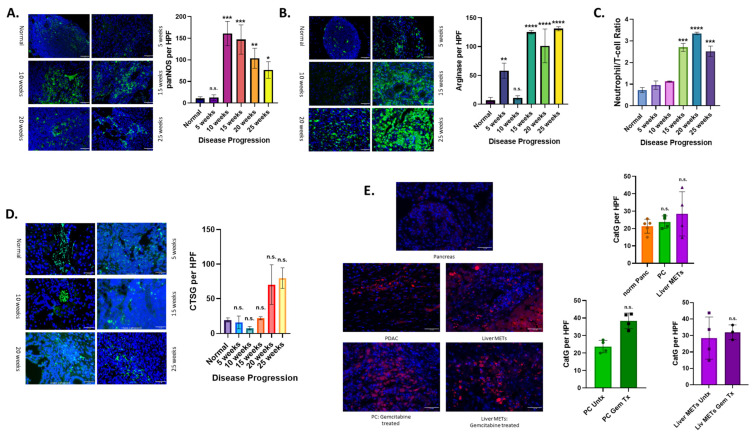
PC-dependent alterations in neutrophil immunomodulation. KPC (K-rasG12D; TP53R172H; Pdx-1cre) pancreatic cancer mouse model samples (n = 3) were analyzed for immunomodulatory markers: panNOS (**A**), arginase (**B**), and CTSG (**D**). PanNOS is an indicator of anti-tumor function and is found to decrease in later stages of disease after an initial increase (**A**). Arginase increased with disease progression and indicated enhanced pro-tumor function (**B**). We tested tissue samples for the presence of both neutrophils (MPO) and T-cells (CD3) to determine if arginase expression affects T-cell recruitment (**C**). With disease progression, there is an increase in the ratio of neutrophils to T-cells (Neutrophils/T-cells), indicating increased immune suppression. Cathepsin G was examined using the CTSG marker in both mouse (n = 3) (**D**) and human samples (n = 20) (**E**). Increased cathepsin G expression with disease progression was seen, indicating increased inflammation in mouse samples. A general trend towards increased inflammation was observed in patient samples when comparing normal samples to the primary tumor, and even more so in liver METs. This was also seen to increase when comparing primary tumors untreated with those treated with gemcitabine. Statistical analysis was done in GraphPad Pro via a non-parametric Mann–Whitney *t*-test or One-Way ANOVA. Scale Bars: 50 µm. *p*-value (* ≤ 0.05, ** ≤ 0.01, *** ≤ 0.001, and **** ≤ 0.0001); n.s., non-significant.

Using the KPC mouse model, we tested several neutrophil-derived immunomodulatory factors to see how they changed with disease progression. We first tested tissue samples with panNOS antibody, which binds to all forms of nitric oxide synthetase (NOS) enzymes, as high expression levels are typically associated with anti-tumor neutrophils (Figure 4A). There was an initial increase in the expression of panNOS at 10 weeks that decreased as the disease continued to progress. Additionally, we analyzed the expression of arginase. Arginase levels are seen to rise with disease progression in our KPC mouse model (Figure 4B).

To determine the effect of the increasing levels of arginase, we used immunofluorescence to look at T-cells through a CD3 antibody. These counts were then compared with neutrophil counts to create a neutrophil to T-cell ratio (Figure 4C). As the disease progresses, we see an increase in the ratio of neutrophils to T-cells. We know from the literature that a high NLR is associated with poor prognosis in patients. We can determine from this data that neutrophils are taking on a more pro-tumor and immunomodulatory function in the TME of PC as disease progresses.

Neutrophils not only have a role in modulating the immune system but also in regulating inflammation. Neutrophils can be pro- or anti-inflammatory depending on the context. Therefore, we tested levels of cathepsin G, an inflammatory molecule produced primarily by neutrophils, to determine what role neutrophils play in the TME. We used Ctsg, a cathepsin G antibody, in both the KPC mouse model and patient samples to determine levels of inflammation (Figure 4D,E). In our KPC mouse model, there is an increase in the expression of cathepsin G with disease progression (Figure 4D). We see a trend towards increased expression when you compare liver METs from patient samples to normal and primary tumor patient tissue samples (Figure 4E). In addition, CTSG expression was enhanced following gemcitabine treatment in primary patient tumors (Figure 4E). This data shows a context-dependent immunomodulatory and inflammatory role for neutrophils in PC TME.

### 3.5. Expression of CXCR1/2 and Its Ligands Is Associated with Poor Survival

**Figure 5:**
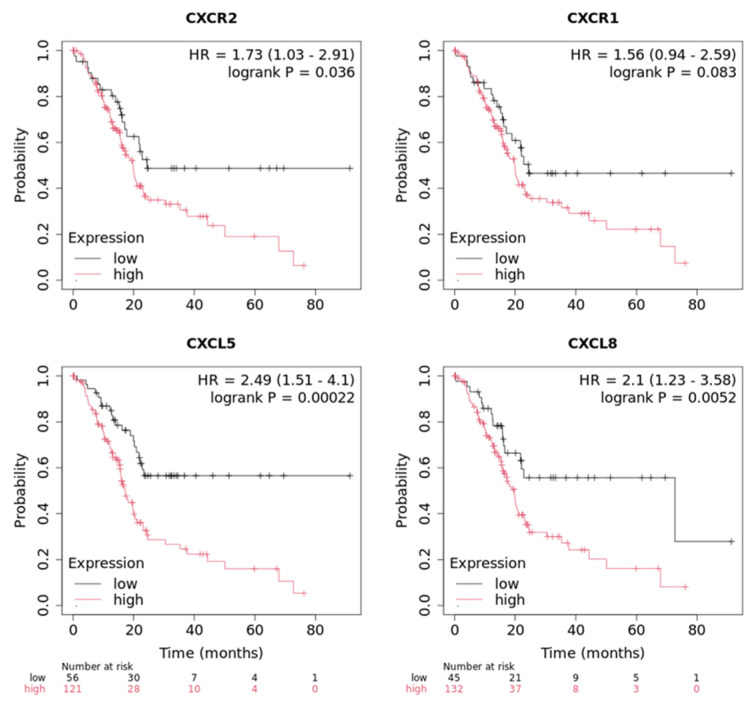
The CXCR1/2 axis diminishes patient survival. Kaplan–Meier plotter, a database of gene profiles from patient samples and their association with patient survival, was used to determine the relationship of CXCR1/2 and their ligands (CXCL5 and CXCL8) to patient survival. Those seen to have high expressions of CXCR2, CXCL5, and CXCL8 had worse rates of survival than those with lower expression.

There is considerable evidence supporting the role of the CXCR1/2 axis in PC progression and therapy resistance from previous research from our lab [51,52,54,55,56,57]. Though it is only partially responsible, the CXCR2 axis aids in neutrophil recruitment to the TME (Figure 2C). Therefore, we used an online diagnostic tool, the Kaplan–Meier plotter, that compares low and high expression of certain genes with patient survival dependent on solid tumor types, to determine how the CXCR1/2 axis affects PC patient survival rates (Figure 5). When patients express high levels of CXCR2 we see a large, statistically significant (*p*-value < 0.05) decrease in the probability of survival (Figure 5). CXCR1 showed similar trends but the expression was not statistically significant (*p*-value > 0.05), likely because of better survival of some patients in later months. We also wanted to see how the expression of CXCR2 associated ligands, CXCL5 and CXCL8 (also a CXCR1 ligand), altered patient survival (Figure 5). We observed similar results to CXCR2, with the survival probability of high expressing patients at 20 months being approximately 20–25% for CXCL5 and 30% for CXCL8 and dropping significantly with no patients surviving in the past 80 months. Low-expressing patients maintained a 55–60% survival, with one patient surviving past 80 months for CXCL5, and CXCL8 showed similar results but had a large drop in survival after 60 months to a 20% survival, though one patient still survived past 80 months. This data was statistically significant (*p*-value < 0.001 for CXCL5 and *p*-value < 0.006 for CXCL8) and shows the importance of CXCR2 and its ligands to patient survival.

## 4. Discussion

In this study, we observed a progressive increase in neutrophil infiltration and NET formation during disease progression, using both murine models and human pancreatic cancer specimens. CXCR2 and its ligands played a central role in mediating neutrophil recruitment and NET generation. Notably, gemcitabine chemotherapy further amplified these effects. As the disease advanced, neutrophils exhibited an increasingly immunosuppressive phenotype. Elevated expression of CXCR2 and its ligands consistently correlated with poor survival in pancreatic cancer patients.

Patients with PC who have a high NLR in their circulating blood are shown to have a worse prognosis than those with a lower NLR [40,58]. We have shown that neutrophils are more abundant in primary tumors, even more so than in liver METs, and that those neutrophils are not all becoming NETs. This indicates that neutrophils are necessary for the primary tumor. The premise is underscored by two separate genetically engineered mouse models of PC that show neutrophil infiltration increases with disease progression in primary tumor sites. We have also revealed that NET formation increases with disease progression in our mouse model, likely preparing primary mouse tumors for metastasis. We observed enhanced levels of NET formation in both our patient models and liver metastasis (MET) samples, which only had slightly elevated neutrophil infiltration, when compared to normal patient samples, highlighting the need for NETs in metastasis and setting up a premetastatic niche, which has been previously established in our laboratory [59]. We have concluded from this data that neutrophils are recruited to the TME of PC to aid in disease progression and metastasis.

Previous research from our laboratory has implied the CXCR1/2 axis in the progression and development of chemotherapy resistance in PC tumors [51,52]. Genetic comparison of normal tissue, primary tumors, and metastasis has shown that patients have enhanced expression of CXCR2 ligands in both metastatic and primary tumors. This data was consistent in our PC patient samples and, additionally, showed increased CXCR2 ligand expression in our primary tumors post-gemcitabine treatment. When we used our KCC mouse model, with Cxcr2 KO, we saw decreased tumor size and diminished number of infiltrating neutrophils. We also observed NETs and neutrophils in the TME of PC patient samples post-gemcitabine treatment, and in both the primary tumor and liver METs, there was an increase in the permeation of neutrophils and development of NETs. While this could be attributed to changes in inflammation, in combination with previous research from our laboratory and literature, this does give weight to our hypothesis that neutrophils may be playing a role in gemcitabine chemotherapy resistance in PC [52,54,55,56,57,60,61]. This data highlights the possible multipurpose role neutrophils play in the TME, beyond their inflammatory and immunomodulatory roles.

PC takes advantage of neutrophils’ immunomodulatory and inflammatory mechanisms. Our KPC mouse model was used to analyze the inflammatory and immunomodulatory function of neutrophils in PC. We saw the modulation of panNOS which can be used to look at levels of all NOS, including iNOS. In the past, iNOS has been associated with an anti-tumor signature in neutrophils; however, in more recent works, iNOS has tumor-benefiting functions at low expression levels, and inhibition has been shown to aid in tumor control [62,63]. Our data indicate that higher levels of iNOS may be important for tumor initiation, but that the expression decreases with disease progression. With this, we observed increased arginase expression with disease progression and an associated increase in the neutrophil-to-T-cell ratio in the TME of mouse tumors. Arginase has been shown to play a significant role in T-cell regulation, including increasing the suppressive activity of T-regs, decreasing CD4+ and CD8+ T-cell proliferation, and decreasing T-cell activation [64,65,66]. This shows that neutrophils are taking on a pro-tumorigenic and immunosuppressive phenotype in the PDAC TME. One major caveat of this study is that some of the iNOS signaling may be due to other immune cells in the TME, such as tumor-associated macrophages (TAMs). However, we observe enhanced iNOS expression in our neutrophils upon PC CM treatment, suggesting that neutrophils are at least partially responsible for the elevated iNOS levels in the TME. However, more research is warranted in this area.

PC tumors take advantage of neutrophils’ ability to control T-cell recruitment [67,68]. We demonstrate that there is a large increase in the production of arginase in the TME of our KPC tumors. Increased ratios of neutrophils to T-cells in the TME as disease progresses in these models, in addition to the enhanced arginase levels, indicate that these neutrophils are inhibiting the infiltration of anti-tumor T-cells. This data simulates a high NLR, with a higher score indicating worse survival [40].

Cathepsin G was used in our study to evaluate inflammation levels in both our KPC mouse model and patient samples. In mouse models, we detected increased inflammation with disease progression and enhanced inflammation in our gemcitabine-treated patient samples. While in some cancers, inflammation is found to be adverse for tumors, in PC, it has been shown that inflammation benefits PC, including aiding in gemcitabine chemotherapy resistance [8]. PC’s ability to tightly control the inflammatory functions of neutrophils is critical to the success of tumors and has been shown in the literature before [69,70,71].

Patient survival can be impacted by several factors; we used an online dataset to analyze the expression of several neutrophil chemoattractants and how they relate to patients’ survival. High expression of either CXCR1 or CXCR2 was shown to be worse for patient outcomes, but CXCR2 was seen to be statistically significant to their prognosis in addition to two of its ligands, CXCL5 and CXCL8, with CXCL5 being more statistically significant than CXCL8. This indicates that overall, these neutrophil chemoattractants are worse for patients in terms of disease progression and survival.

Overall, our data demonstrate that neutrophils are actively recruited into the TME of PC, where they play key roles in immune suppression and inflammation that the tumor exploits. Their presence is further amplified by chemotherapy, suggesting a role in the development of therapy resistance, potentially through inflammatory mechanisms. Neutrophils support both primary tumor growth and metastasis and are associated with poor prognosis in patients. These findings underscore the potential benefit of therapies that inhibit neutrophil recruitment or reprogram their pro-tumor functions to be anti-tumor. However, further studies are needed to elucidate how PC reprograms neutrophils toward a tumor-promoting phenotype.

## 5. Conclusions

This study, using both murine models and human pancreatic cancer specimens, concludes that there is a progressive increase in neutrophil infiltration and NET formation during disease progression in PC TME. CXCR2 and its ligands played a central role in mediating neutrophil recruitment and NET generation. Notably, gemcitabine therapy further amplified these effects. As the disease advanced, an increasingly evident immunosuppressive phenotype was exhibited by neutrophils. Elevated expression of CXCR2 and its ligands consistently correlated with poor survival in pancreatic cancer patients.

## Figures and Tables

**Table 1:** Antibodies in this Study.

No.	Human/Mouse	Primary Antibody	Marker	Source	Catalog No.	Dilution	RRID
1	Human	Anti-myeloperoxidase	Neutrophils	Thermo Scientific, Fremont, CA, USA	RB-373-A0	1:200	AB_59598
2	Human	Cathepsin G	Inflammation	Santa Cruz Biotechnology, Santa Cruz, CA, USA	sc-33206	1:200	AB_2087522
3	Human	Histone H2A	NETs	Santa Cruz Biotechnology, Santa Cruz, CA, USA	Sc-8648	1:200	AB_2118018
4	Human	IL8	CXCL8	Pierce Endogen, Rockford, IL, USA	M802B	1:200	AB_223584
5	Human	CXCL5 ELISA capture antibody	CXCL5	R&D Systems, Minneapolis, MN, USA	DY254-05	1:500	AB_2245472
6	Mouse	Anti-myeloperoxidase	Neutrophils	Abcam, Waltham, MA, USA	ab9535	1:200	AB_307322
7	Mouse	Cathepsin G	Inflammation	Santa Cruz Biotechnology, Santa Cruz, CA, USA	Sc-6514	1:200	AB_2087408
8	Mouse	Citrullinated Histone H3	NETs	Abcam, Waltham, MA, USA	Ab5103	1:200	AB_304752
9	Mouse	CD3e	T-cells	BD Pharmogen, Franklin Lakes, NJ, USA	553057	1:50	AB_394590
10	Mouse	Arginase	Arginase	Santa Cruz Biotechnology, Santa Cruz, CA, USA	Sc-271430	1:10	AB_10648473
11	Mouse	panNOS	NOS	Santa Cruz Biotechnology, Santa Cruz, CA, USA	Sc-58399	1:10	AB_784875

## Data Availability

The data presented in this study are available in the article published.
